# Consistently Inconsistent Perceptual Illusions in Nonhuman Primates: The Importance of Individual Differences

**DOI:** 10.3390/ani13010022

**Published:** 2022-12-21

**Authors:** Michael J. Beran, Audrey E. Parrish

**Affiliations:** 1 Department of Psychology and Language Research Center, Georgia State University, Atlanta, GA 30302, USA; 2 Department of Psychology, The Citadel, Charleston, SC 29409, USA

**Keywords:** illusions, individual differences, vision, primates, monkeys, comparative cognition, perception

## Abstract

**Simple Summary:**

Visual illusions fascinate humans, in large part because we realize how such experiences disconnect how we perceive the world from reality. The discovery that other animals also experience some of these illusions has provided a compelling comparative story about the role that perception plays in sometimes misrepresenting the nature of the real world. What has also become apparent from comparative studies is that not all animals experience illusions the same way, and sometimes the same individual may not experience some illusions while experiencing others. A survey of the results of 14 experiments from 10 different studies with two monkey species that performed numerous illusion tasks reinforces the idea that individual differences are a rich source of information for better understanding any universal principles of visual perception. Future research should focus more effort towards understanding the causes and effects (on other behaviors) of experiencing (or not experiencing) these illusions.

**Abstract:**

Perceptual illusions, and especially visual illusions, are of great interest not only to scientists, but to all people who experience them. From a scientific perspective, illusory visual experiences are informative about the nature of visual processes and the translation of sensory experiences to perceptual information that can then be used to guide behavior. It has been widely reported that some nonhuman species share these illusory experiences with humans. However, it is consistently the case that not all members of a species experience illusions in the same way. In fact, individual differences in susceptibility may be more typical than universal experiences of any given illusion. Focusing on research with the same nonhuman primates who were given a variety of perceptual illusion tasks, this “consistent inconsistency” is clearly evident. Additionally, this can even be true in assessments of human illusory experiences. Individual differences in susceptibility offer an important avenue for better understanding idiosyncratic aspects of visual perception, and the goal of isolating any possible universal principles of visual perception (in primates and beyond) should address these individual differences.

## 1. Introduction

Perceptual illusions fascinate humans. They are omnipresent in the modern world. Visual illusions are popular on social media platforms, where there are yearly articles on the best illusions from that year [1], and where one finds endless debate about what people think they may see, and why it is so fascinating (and frustrating) that others do not see the same thing (e.g., is the dress blue or gold? [2]). Beyond entertainment, illusions are often featured in scientific settings for the public (for example, the Fernbank Museum of Natural History in Atlanta has featured an exhibit on sensory experience and perceptual illusions for many years), and they are even a part of the educational system for many children. In a truly fortuitous occurrence, the same day that the first author was working on this paper, his 12-year-old daughter had a school assignment that featured illusions such as the Müller-Lyer illusion [3], the Thatcher Effect [4], and the Rotating Snake Illusion [5]. Her assignment was, first, to experience these illusions, and then to write about why they occur, why they may be adaptive, and what they tell us about the nature of sensory experiences and how those may differ from perceptual processes. Her father helped her a little with this homework, of course, including by explaining to her who Margaret Thatcher was! As part of this, it was interesting to see that not all illusions worked equally well on both of them, which is an anecdote highly relevant to the main point of this article.

Perceptual illusions serve a purpose beyond entertaining us as we experience them. They help us understand the constraints of our perceptual experiences, and they inform us about how other cognitive mechanisms interact with sensation and perception to generate such experiences [6]. Although perceptual illusions are “errors in the machine,” they also are tools for understanding how that machine works more broadly, in the same way that decision-making errors and biases inform us about how we frame decisions and calculate the value of options. The ancient Greeks considered the causes and implications of perceptual illusions (e.g., Aristotle and the waterfall effect), and the first scientific description of an illusion was carried out by Necker [7], followed by numerous other descriptions of illusions shortly after [8,9]; for a review, see [10]. Central to many of these early experiments was an appreciation of individual differences in how people experienced illusions, although formal assessments of what accounted for those differences often was not undertaken. 

Given this widespread interest in perceptual illusions in our own species, combined with our fascination about the minds of other animals, it is no surprise that studying illusory experiences across species has been a longstanding area of focus in comparative psychology and ethology [11,12,13,14,15,16,17,18,19,20,21,22,23,24,25,26]. Although studies of illusory experiences have been conducted with many species, we will focus our attention on nonhuman primates, simply for the reason that we know more about those species than others, and because nonhuman primates have been among the most extensively tested species. We will be highlighting specific studies from our laboratory that included tests with rhesus monkeys (*Macaca mulatta*) and capuchin monkeys (*Sapajus apella*), sometimes in comparison to nearly identical tests given to human adults and human children. The goal in focusing on that work is not to downplay or ignore the many other creative and informative studies of other primates or non-primate species [11,12,13,14,15,16,17,18,19,20,21,22,23,24,25,26], but to show that even in a restricted sample, some important points emerge about the need to assess and try to account for individual differences in these illusory experiences.

That is the heart of what we want to convey in this article—that we have reached a point in this area of comparative research at which we should begin to study such individual differences more intensively and carefully. Those differences are present and are often acknowledged and reported in empirical studies, even if there is little attempt made to explain why those differences exist. Some of the earliest papers in comparative psychology made this clear. For example, when chicks were given four tests of different illusions, it was noted in the abstract of that report that marked individual differences in susceptibility were shown for all four of the illusions [11]. However, there was no indication of what accounted for these differences. 

Such individual differences likely offer substantial insight into the nature of perceptual processing mechanisms, not for their universality of operation, but for the way in which those mechanisms interact with variables such as species membership, age, sex, foraging ecology, attentiveness, and the prioritization of so-called top-down versus bottom-up processing of stimulus properties. Although there is still excellent value in charting what new species may be susceptible to different illusory experiences, so as to broaden our phylogenetic map of such experiences, there is equal value in trying to understand why some individuals experience illusions whereas others do not. Additionally, there is great value in understanding why *even the same individual* may be susceptible to some illusory experiences but not others. We acknowledge at the outset that we do not (yet) have many answers about this, and so we will instead focus on presenting the nature of the “problem” with the goal of convincing the reader that individual differences offer a rich source of information that can lead to a fuller understanding of perceptual processes as they manifest across species.

Human perception research already acknowledges the importance of understanding the causes and implications of individual differences, and this has been true for many decades [27]. Research with humans has shown that there are cross-cultural differences in illusion susceptibility (e.g., [28,29,30]), that variance in the susceptibility to visual illusions may be linked to individual differences in perceptual processing mode (global vs. local processing [31,32,33,34]), that visual illusory experiences emerge and grow stronger through development [35,36], and that there are often weak inter-illusion correlations when human participants are given numerous illusory tests e.g., [37,38,39]. For example, in one study, 490 observers were given 26 illusions as well as tests of spatial ability. Large individual differences were found with regard to susceptibility to the different illusions. Although these individual differences were related to the assessments of spatial abilities, the direction of that relation was not consistent across the illusions, suggesting that this relationship was not a simple one [40]. Additionally, the authors of that study concluded that “individual differences in the susceptibility to various visual–geometric illusions may be indicative of individual differences in the cognitive processing of perceptual and spatial information in general” [40] (p. 218).

From our own laboratory, we can see evidence of individual differences in nonhuman animals by comparing the performances of monkeys across a variety of tasks designed to document (if present) illusory experiences. This is largely a qualitative description of the degree to which each individual primate showed or did not show the typical (i.e., human-like) pattern of misperceiving stimuli as they truly exist, instead falling prey to perceptual experiences that were misaligned with sensory inputs from the visual systems of these animals.

## 2. Materials and Methods

Data were drawn from published papers from the Language Research Center at Georgia State University that included rhesus monkeys and/or capuchin monkeys, and that specifically focused on one or more geometric visual illusions. We included any papers that had one or more primate subjects in them who also were subjects in at least three other experiments from our laboratory that assessed perceptual illusion susceptibility. Any included papers had to report the specific performances of each subject with regards to the illusory outcome (e.g., misperceiving item size or relative quantity), so that we could assign a positive or negative value to that subject for that illusory experience. We chose to classify each subject as either “showing the illusion” or not, rather than trying to quantify the magnitude of the illusion, because the latter effort would have excluded a large number of these papers, and we wanted a larger sample size. This resulted in a total of 10 studies and 14 individual experiments, each of which is described below. Across all experiments, a total of 8 rhesus monkeys and 10 capuchin monkeys met criterion for inclusion in this summary by participating in at least four experiments.

**Figure 1:**
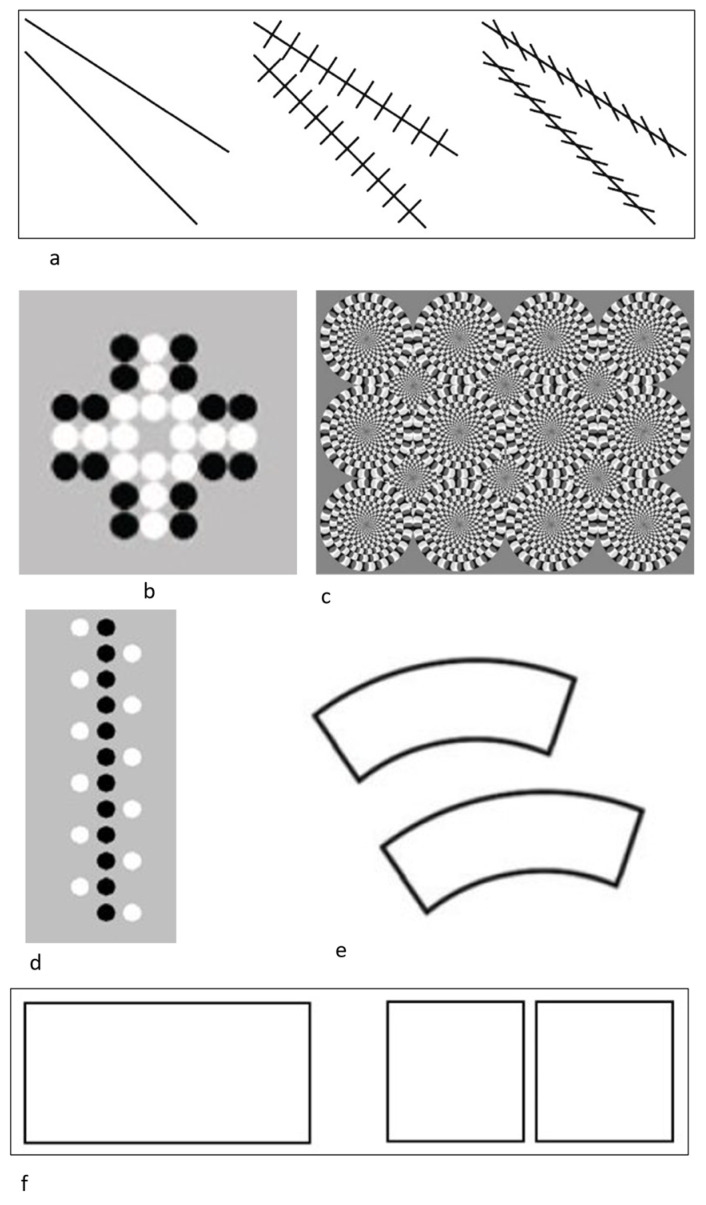
The illusions presented to monkeys, including the (**a**) Zöllner illusion with control stimuli at left, non-illusory cross-hatched stimuli at center, and the illusory image at right; (**b**) Solitaire illusion in which centrally located white dots are overestimated relative to an equal number of black dots on the perimeter; (**c**) Rotating Snake Illusion in which the static circular patterns appear to rotate; (**d**) density bias illusion in which the central, contiguous array of black dots appears more numerous than an equal number of white dots on the periphery; (**e**) Jastrow illusion in which the lower stimulus is typically reported to look longer than the upper stimulus; (**f**) one-is-more illusion in which the continuous stimuli at left appears larger than its partner discrete stimulus at right.

*Agrillo et al. (2014a)*: To assess possible susceptibility to the Zöllner illusion (Figure 1a), rhesus monkeys were trained to select the narrower of two gaps at the end of two convergent lines presented on a computer screen. After training, three conditions were presented. In two control conditions, there were no crosshatches present or those crosshatches were perpendicular, and so no illusory experience could occur. In the illusory condition, the crosshatches were aligned in the manner that induced the illusion in humans (i.e., oblique crosshatching lines). The results showed that rhesus monkeys perceived the Zöllner illusion in the same direction as humans [41].

*Agrillo et al. (2014b)*: Rhesus monkeys and capuchin monkeys were assessed for their susceptibility to the Solitaire illusion (Figure 1b, [42,43]). The task involved choosing one of two arrays based on that array having more white dots. The Solitaire configuration (clustered central dots forming a better Gestalt) typically leads to overestimation relative to the same number of dots located on the periphery of the array. Approximately half of the monkeys showed some susceptibility to this illusion in the first experiment, but there were substantial individual differences. An attempted replication within the same study with the same monkeys largely failed, providing evidence that this illusion was fleeting, at best, in these species.

*Agrillo et al. (2015)*: This experiment assessed whether rhesus monkeys experienced illusory motion using the Rotating Snake Illusion (Figure 1c; [44]). Real motion is perceived based on a specific luminance pattern comprising concentric circles (black–dark grey–white–light grey). Monkeys were first trained to choose dynamic arrays with moving stimuli over static arrays. They then were given a choice between the Rotating Snake Illusion and a control image that was highly similar but alternated color patterns in a way that does not produce the experience of illusory movement for humans. In a second variation of the task, monkeys saw a single stimulus and had to classify it as having movement present or not. In training trials, real movement was present, and in test trials with the Rotating Snake Illusion, the question was how the monkeys would classify that stimulus. Some monkeys responded in a manner consistent with experiencing illusory motion, although the effect was subtle relative to some other illusory experiences. A second experiment required the monkeys to learn to choose the Rotating Snake stimulus over a static stimulus, and then compared performance to when two static stimuli were presented, and one was the reinforced choice. Most monkeys performed better and learned faster when asked to choose the Rotating Snake image, again suggesting some experience of illusory motion.

*Parrish et al. (2015)*: In this study [45], monkeys and adult humans were tested on the Delboeuf illusion, in which small concentric rings lead to the overestimation of central dot size (assimilation effects) and large concentric rings lead underestimation of central dot size. Rhesus monkeys and capuchin monkeys were trained to select the larger of two black dots encircled by large and small concentric rings in Experiment 1. Adult humans and several monkeys perceived the illusion in the standard direction, but the majority of monkeys perceived a reversed illusion (selecting the dot encircled by the large ring as larger) or did not perceive the illusion in either direction. To rule out an alternate explanation (that the monkeys were responding on the basis of outer ring size vs. central dot), we trained monkeys to classify a central dot that was encircled by an outer ring of variable size as ‘small’ or ‘large’ in Experiment 2. Adult humans and the majority of monkeys perceived the illusion in the standard direction, underscoring the importance of methodological design in the study of visual illusions.

*Parrish et al. (2016)*: In this experiment [36], we presented the Solitaire illusion (see Figure 1b) as described above via computerized testing to preschool children and task-naïve capuchin monkeys that had limited experience with computerized testing to further explore the role of experience in the emergence (or not) of this illusion. The task-naïve capuchin monkeys perceived the illusion, but there were large individual differences in susceptibility akin to our previous results [43]. Younger children performed similarly to monkeys in terms of the variance in illusion susceptibility, whereas older children were more consistent in their perception of the Solitaire illusion. Furthermore, individual susceptibility by capuchin monkeys to the Solitaire illusion did not correlate with a related numerosity illusion, the density bias [46,47].

*Parrish et al. (2019)*: In this experiment studying linear numerosity illusions (a variant of the Solitaire array described above), rhesus monkeys and capuchin monkeys were trained to choose the array with a larger number of black dots relative to white dots. The illusory stimuli were those in which the central, contiguous array was black, whereas the white dots were peripheral to that central arrangement (Figure 1d). Humans tend to perceive the central, linear array as being more numerous than an equal number of dots on the periphery as the central dots form a better Gestalt. Approximately half of the monkeys showed the same illusory effect, although it was subtle [48]. Additionally, again, there were substantial individual differences, with one monkey showing a reverse bias to that seen in humans.

*Agrillo et al. (2019)*: This study assessed the Jastrow illusion (Figure 1e) in capuchin monkeys and rhesus monkeys [49]. In this illusion, humans typically overestimate the size of the bottom figure relative to the identically sized top figure due to their spatial layout. Despite learning that they needed to select the larger of two stimuli in a computerized two-choice discrimination task, none of the monkeys we tested showed susceptibility to this illusion when identically sized images were arranged in the Jastrow pattern [50]. Susceptibility to the illusion may be supported by global processing mechanisms, which emerge more readily for human subjects relative to nonhuman primates.

*Parrish et al. (2020)*: This experiment assessed the density bias in capuchin monkeys using arrays of food as the choice options [46]. This bias emerges when densely arranged stimuli are overestimated or preferred relative to an equal number of sparsely arranged items. Monkeys saw arrangements of food items in which those items were sparsely distributed or densely arranged. Most monkeys were biased to select a denser food set over the same number of food items in a sparsely arranged set, suggesting that they misperceived those items as being numerous. These results were consistent with computerized testing of the density bias with these same monkeys [47], although those results are not included in the current review as individual performances were not reported.

*Parrish and Beran (2021)*: In this experiment, rhesus monkeys were trained to choose the larger of two rectangular stimuli on a computer screen. After becoming proficient at this, they were given trials in which either of both choice options were of low contrast to the background white color (i.e., gray rectangles) or were of high contrast (i.e., black rectangles). In other trials, the background was black, and so white rectangles were of high contrast and gray rectangles were again lower contrast. For humans, higher contrast stimuli are often overestimated in terms of their size [51], likely due to perceptual fluency of high-contrast relative to low-contrast stimuli. This result was found for some of the monkeys as well [52].

*McKeon et al. (2022)*: Rhesus monkeys were presented with the “one is more” illusion [53] to determine whether they would show a comparable illusion to that recently reported in humans [54]. When presented with continuous objects rather than multiple discrete objects, humans experience the continuous objects as being longer compared to the discrete objects of equal length. Monkeys were trained to choose the longer of two truly different-length items on a computer screen, and then they were given probe trials in which a continuous stimulus and a discrete stimulus of equal length were presented (Figure 1f). Unlike humans, the monkeys showed no preferences in these trials, and overall they performed very highly by ignoring the discrete/continuous relation and instead focusing on the true length of stimuli to make judgments.

## 3. Results

Table 1 shows the results of this qualitative survey. Not a single monkey was susceptible to all illusory stimuli to which they were exposed. At the other extreme, one monkey (Hank) showed susceptibility in only one of the nine experiments in which he participated. All other monkeys showed susceptibility to some but not all illusions, and many of them showed susceptibility about half of the time. For those studies in which two experiments were conducted, providing a conceptual replication within-study, there were also changes in susceptibility for the monkeys. In Agrillo et al. [43], only 1 of 13 monkeys replicated their susceptibility in both experiments. In Agrillo et al. [44], only two of six monkeys showed the illusory effect in both experiments. In Parrish et al. [45], only one monkey (Gale) showed the illusory effect in both experiments, and in Parrish and Beran [52], three of five monkeys replicated the illusory outcome. These qualitative results make clear the variability across illusions within this sample of monkeys, and also the variability within individuals not only for different illusions, but also for the same illusion tested at different times. 

**Figure 2:**
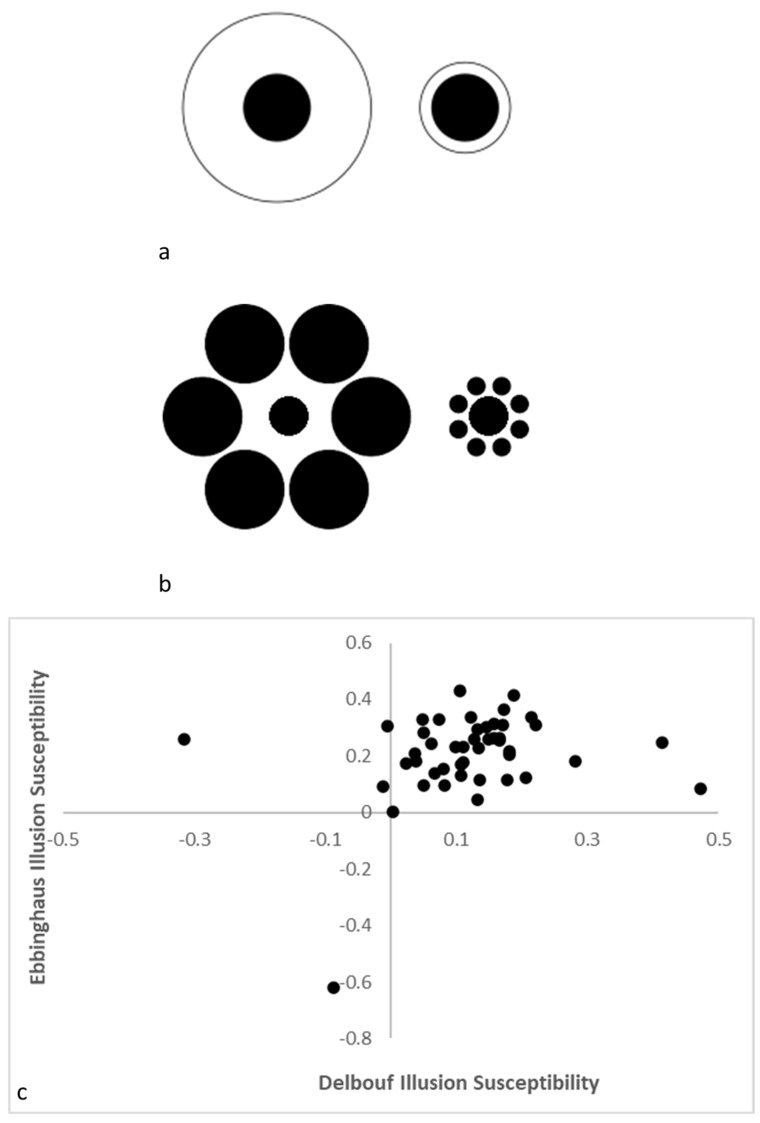
The Delboeuf (**a**) and Ebbinghaus (**b**) illusions in which the central dots surrounded by a smaller perimeter ring or a series of smaller other dots (i.e., the right stimuli in each image) are overestimated relative to when the surrounding ring or series of circles are large. The relative susceptibility to each of these illusions in a sample of 46 adult humans where positive values indicate the typical illusory experience (**c**).

In an unpublished study from our laboratory conducted with adult humans (college students at Georgia State University), we can see a similar pattern. In that study, we presented 46 participants with the Delboeuf illusion (hereafter DBI; [55]) and the Ebbinghaus illusion (hereafter EBI; [56]) in the context of having those participants adjust an onscreen dot until they thought it matched the size of a comparison stimulus presented in an illusory context (Figure 2a,b). Although most participants had a tendency to overestimate the size of stimuli presented in a small surrounding circle for the DBI and centered among small surrounding circles in the EBI, there were individuals who did not show this typical susceptibility. The relation of the magnitude of DBI and EBI susceptibility in this sample was nonsignificant, r(44) = 0.23, *p* = 0.12, and it accounted for only 5.3% of the variance in the sample (Figure 2c). As with the monkeys, humans are variable in their susceptibility to closely related illusory experiences, even within the same task given at the same time to the same people. 

## 4. Discussion

The take-home message of this brief survey of a sample of primates, given a moderate range of perceptual illusory experiences, is that there was a “consistent inconsistency” in their susceptibility to such illusions. Across these studies, rarely did any specific primate experience most or all illusions, nor did any specific animals experience few or none of the illusions. Rather, the vast majority of these animals sometimes responded as if they experienced a given illusion, although across multiple tests of the same or similar illusions, there was variability at the level of the individual. Additionally, this pattern matched that of an unpublished sample of human participants given two well-known illusions, the Delboeuf illusion and the Ebbinghaus illusion, even though those two illusions were presented within the same task, using the same computerized apparatus. It also matched the consistent reports of large-scale studies with humans in which there were large individual differences in susceptibility [27,37,38,39,40].

There are a number of reasons why illusory experiences in nonhuman animals (and humans) may be consistently inconsistent. One strong candidate for accounting for difference across illusions (and studies) is methodological variation. How individuals are trained to perform tasks, how stimuli are created and presented, and what form responses take in tasks all could affect inconsistencies in experiencing illusions across studies (see [57,58,59]). Recognizing the relative priority of global versus local modes of processing visual stimuli in different species and individuals also can account for variation in illusion susceptibility (e.g., [60,61,62,63,64,65,66]). Differences in psychophysical discriminability capacities also are relevant, as are factors such as discrimination thresholds (acuity in size and numerosity discriminations), motivation to engage with tasks, previous testing histories that may conflict with or support a more illusory experience in newer tasks, and even factors such as how quickly or slowly individuals (and species) respond in tasks. It is likely that all of these factors and others play a role in the species differences and individual differences that are evident in the literature. In some cases, relevant factors could be controlled in ways that better isolate illusion susceptibility, although it is important to also assess illusory experiences in more naturalistic choice and decision setting (e.g., chimpanzees falling prey to the DBI when being allowed to look at and choose food items to immediately consume [67]). 

Although we cannot (yet) adequately explain the individual differences seen in our monkeys, or other animals, this is an attainable goal, and will allow a productive focus of future research. There is a richness in these individual differences for better understanding the cause(s) of illusory experiences, and more broadly for understanding the different phenomenal worlds of different species, different individuals within a species, and the same individuals tested at different times. Although there are certainly major differences in how some species may perceive and process sensory information relative to other species (e.g., the bat versus the reptile), there may also be substantial intraspecific differences that affect other behavioral and cognitive processes. As just one example, based on some of the studies shown in Table 1, differences in susceptibility to numerical or quantitative illusions could impact foraging behavior, social approach or avoidance behavior, and other natural behaviors that rely on perceiving and representing magnitude information such as quantity or number. The same is true for size-based judgments, which may be affected by those individuals who are more susceptible to illusory experiences. 

Most likely, those individual differences do not lead to substantial fitness benefits to those who are less susceptible to illusions, or else there would have been selection against such susceptibility. However, this does not mean that illusory experiences are not detrimental. It would be very informative to add illusion susceptibility to some of the “cognitive testing batteries” that have been developed for nonhuman primates e.g., [68,69] and adapted for other species e.g., [70]. One could predict that illusory experiences may generate more decision errors in some kinds of choice tasks, or greater likelihood of errors in different kinds of memory tests, and especially those for which one must remember the actual attributes of a stimuli that could be misrepresented due to the illusory experience. As such, documenting and accommodating individual differences in illusory experience could aid in accounting for variance in animal cognition and behavior that has otherwise not been accounted for through the manipulation of other factors. 

## 5. Conclusions

Comparative psychology has provided convincing evidence that other species experience many of the same perceptual illusions that humans experience. This is particularly true for nonhuman primates, which makes sense given their evolutionary kinship with humans. However, as is also true for humans, such illusory experiences are not universal, and there are individual differences in the perception of any given illusion as well as inconsistent illusory experiences of the same individual when shown different illusory stimuli. This “consistent inconsistency” is fertile ground for better understanding the nature of perceptual experience, for understanding the mechanisms at work during illusory experiences, and the broader degree to which such experiences may (or may not) affect other cognitive and behavioral mechanisms that rely on and respond to perceptual experience (whether illusory or not). Future comparative research should focus more on individual differences, toward the goal of providing new insights toward understanding the different phenomenal world of diverse species. These insights may also contribute to important practical considerations regarding how we maintain and care for primates in captivity. As we increasingly recognize that individual differences exist among primates, and that different primates can have their own unique perceptual experiences, this could affect how we care for those animals in a way that is more beneficial. 

## Figures and Tables

**Table 1:** Susceptibility of each monkey in each experiment in terms of experiencing the illusion in the same direction as for humans.

Monkey	Agrillo et al., 2014a	Agrillo et al., 2014b Exp 2	Agrillo et al., 2014b Exp 3	Agrillo et al., 2015 Exp 1	Agrillo et al., 2015 Exp 4	Parrish et al., 2015 Exp 1	Parrish et al., 2015 Exp 2	Parrish et al., 2016 Exp 1	Parrish et al., 2019	Agrillo et al., 2019	Parrish et al., 2020	Parrish & Beran 2021 Exp 1	Parrish & Beran 2021 Exp 2	McKeon et al., 2022
Macaques														
Chewie		YES	NO			NO	YES		YES	No		YES	NO	NO
Gale		YES	NO	YES	NO	YES	YES							
Han		YES	YES			NO	YES		YES	No		NO ^	NO ^	NO
Hank		NO	NO	YES	NO	NO	NO ^		NO	NO				NO
Lou	YES			YES	YES	NO	YES		NO	NO		YES	YES	NO
Luke	YES	NO	NO	YES	YES				NO	NO				NO
Murph	YES	NO	YES	NO	YES	NO	YES		NO	NO		YES	YES	NO
Obi				NO	NO	NO	YES		YES	NO		YES	YES	NO
Capuchins														
Gonzo *						NO	YES	YES	NO	NO				
Gretel *						NO	YES	YES	NO	NO				
Griffin		YES	NO			NO	YES		NO	NO	YES			
Liam		NO	NO			NO ^	YES		NO	NO	YES			
Logan		YES	NO ^			NO ^	YES		YES	NO	NO			
Mason						NO ^	YES	NO	NO	NO				
Nala *		NO	NO			NO ^	NO ^		NO	NO	YES			
Nkima		YES	NO			NO	YES		NO ^	NO	NO			
Widget *		NO	NO						YES	NO	YES			
Wren *		YES	NO			NO	YES		YES	NO	YES			

Note. All rhesus macaques were males, as our laboratory only housed male rhesus monkeys during the period in which these studies were conducted. Female capuchin monkeys are designed with an *. In some cases, monkeys showed a reverse pattern to that seen in humans. These cases are indicated with ^. Additionally, note that we only included in this table those monkeys who were in four or more experiments. In some of these experiments and studies, there were other monkeys who may have shown or not shown the illusion, so susceptibility within a species may be more or less evident when including the full sample. The reader is directed to the specific studies to see the full range of performance of the monkeys in each study.

## Data Availability

All data discussed in this article are included in the article itself, or they are reported in the published papers listed in Table 1.
